# Inferring the Rate-Length Law of Protein Folding

**DOI:** 10.1371/journal.pone.0078606

**Published:** 2013-12-05

**Authors:** Thomas J. Lane, Vijay S. Pande

**Affiliations:** 1 Department of Chemistry, Stanford University, Stanford, California, United States of America; 2 Departments of Chemistry, Computer Science, and Biophysics, Stanford University, Stanford, California, United States of America; Oak Ridge National Laboratory, United States of America

## Abstract

We investigate the rate-length scaling law of protein folding, a key undetermined scaling law in the analytical theory of protein folding. Available data yield statistically significant evidence for the existence of a rate-length law capable of predicting folding times to within about two orders of magnitude (over 9 decades of variation). Unambiguous determination of the functional form of such a law could provide key mechanistic insight into folding. Four proposed laws from literature (power law, exponential, and two stretched exponentials) are tested against one another, and it is found that the power law best explains the data by a modest margin. We conclude that more data is necessary to unequivocally infer the rate-length law. Such data could be obtained through a small number of protein folding experiments on large protein domains.

## Introduction

A deep understanding of protein folding must involve a description of the general mechanisms involved. It is reasonable to suspect this will consist of a simple model, based on microscopic physics, expressed in a simple mathematical language. Such a model would show how biological sequences are able to employ physics to spontaneously self-assemble into intricate molecular machines.

Simple models of this sort often start by postulating a mechanism of folding, and then derive the consequences of that mechanism [1]–[12]. This suggests it might be possible to infer the general mechanisms of protein folding by verifying the specific predictions of these models. For a simple model of protein folding, however, there is a limited set of *general* experimental trends that can be readily predicted. One such experimental trend is the law governing how folding times scale with chain length. This is perhaps the simplest comparison of theory and experiment possible, but has not yet been unambiguously inferred despite nearly two decades of active research [2], [7].

Polymer theory provides strong precedent for using chain-length scaling laws as a point of connection between experiment and theory. In polymer theory, for instance, comparing the theoretically predicted scaling of the longest relaxation time and polymer diffusion constant as a function of polymer chain length demonstrates deficiencies in the classical Rouse model [13]. Inclusion of hydrodynamic interactions – as is done *e.g.* in the Zimm model [14] – is necessary to get the correct scaling laws for these kinetic parameters [15]. Thus, comparing chain-length scaling laws in theory and experiment yields new scientific insight, namely that hydrodynamic effects contribute significantly to polymer dynamics in solution. Our hope is that by determining a rate-length law for protein folding, similar comparisons between experiment and theory will yield new insight into how proteins fold.

The rate-length law is also an interesting result in and of itself. Such a law can be viewed as a statement of the computational complexity of protein folding – given a problem of size 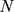 (residues), how does one expect the time-to-solution (folding) to scale? Levinthal pointed out that an exhaustive search would result in exponential scaling, and suggested that this would result in unreasonably large folding times [16]. Thus, in many ways, a resolution to Levinthal's paradox is likely to be phrased directly as a rate scaling law, either non-exponential (polynomial) or exponential with an explicitly small exponential factor.

A number of issues complicate inferring such a law from experiment, most importantly the fact that available kinetic data on protein folding spans a very limited range of chain lengths - about 30 to 300 residues [17]. The statistical power of the data is inherently limited by the fact that protein domain sizes barely span a single order of magnitude, and that most studies of folding have focused on small, well-behaved model systems.

Further complicating the inference of the rate-length scaling law is the fact that chain length is certainly not the only factor affecting folding rates. In fact, it has been argued that it is a fairly weak predictor of the folding time [18], [19]. For instance, there seems to be some correlation of folding times with topological complexity of the native state, such that if two proteins have the same number of residues, but different folds, they may take different amounts of time to fold [18], [20]. Moreover, even protein mutants with the same native structure can have at least 3 orders of magnitude variation in their folding rates [21]. Thus, we expect that experimental data on the scaling of folding time with chain length should be very noisy, and difficult to statistically estimate.

Nonetheless, as we will demonstrate, there does seem to be a significant correlation between the number of residues (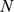) and folding times (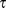). Many different mathematical forms of this scaling law have been postulated, either from theory or empirically, but all fall into one of three basic classes. Shaknovich [2], Cieplak [3], [4], and co-workers have proposed a power-law, 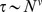. We recently constructed a model that suggested exponential scaling, 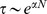 [1], consistent with predictions made by Zwanzig, Szabo and Bagchi [5], [6]. Finally, Thirumalai [7], [8], Muñoz [9], Takada [10], Finkelstein [11], and co-workers have suggested a stretched exponentials, 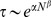, with 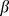 as 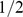 or 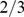. Wolynes has proposed the law may conditionally change between all four suggested models [12].

Each model for the rate-length law derives from a different model of the fundamental physics of folding. Exponential rate laws have been estimated based on models of protein folding as a biased conformational search [1], [5], [6]. Stretched exponentials have been derived by modeling folding as a critical nucleation process [11] or random energy model [7]–[9]. Power laws [2]–[4] and stretched exponentials [10] have been posited empirically based on simulation. Finally, lack of a strong rate-length relationship was suggested based on experimental data [18], [19]. Clearly, different postulates for folding mechanisms lead to different predictions for the rate-length law (or lack thereof). Thus, inferring the rate-length law of protein folding may directly inform our understanding of the mechanisms by which all proteins fold.

In what follows, we develop and apply two methods for choosing between these models and evaluate how each proposed model performs.

## Modeling Methods

Below, we outline two complementary methods for inferring which proposed scaling law is the most reasonable. First, we present a method capable of fitting each model's parameters to the known data, and examining how well each model explains the data. Next, we investigate a second discriminatory method, which proposes that folding times must be below a certain threshold value to be biologically viable. It has been demonstrated that in the crowded milieu of the cell, proteins must fold rapidly to avoid aggregation or degradation. We suggest that this implies that we can check the reasonableness of any model by seeing if its prediction for this threshold time is reasonable with what has been observed empirically in biology.

In this study we focus on single-domain globular proteins. Kinetic data for the folding times of proteins were taken from the KineticDB [17], which reports protein folding times at zero denaturant, near room temperature, and under neutral pH. Other data sets exist [22]–[24], but were not consistent with one another – despite this, they yielded very similar results (Fig. S1, Table S1 in File S1).

### Direct Method: Likelihood Maximization

We want to estimate the parameters for each proposed form of the scaling law. In what follows, we adopt a model that accounts not only for this scaling law, but all other factors (topology, experimental conditions, etc.) via a random Gaussian component. Thus, by fitting each model, we not only learn parameters for each proposed model, but also get an estimate for the relative importance of these other factors in determining folding times.

We assert the following model for the folding time,
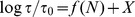
(1)where
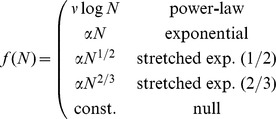
are the proposed folding rate laws, 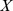 represents a random variable distributed (independently and identically) as a zero-mean Gaussian, 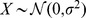, and 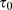 is a fit constant accounting for units of time. These models include the four proposed rate-length laws suggested in the literature and a null model that corresponds to the lack of a rate-length relationship. By adding 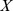 to the logarithm of the folding time (1), we model random variation in *relative* terms, and it enters as a multiplicative factor.

The random variable 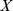 models all contributions to the folding rate not accounted for by the chain length. In a first-principles model, this would possibly include effects due to specific sequences, folded state topology, experimental conditions, or perhaps other factors. Here, we lump these terms into a single random variable in order to focus on the chain length exclusively.

Equation (1) implies 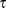 is distributed as a log-normal, with location parameter 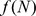 and scale parameter 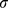. The likelihood of the entire data set (assuming 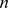 independent measurements) is
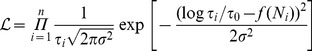
(2)We have three parameters for each model, 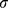, 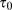, and 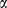 or 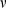 for the exponential and power-law families, respectively.

We have fit these parameters by maximizing the likelihood 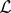. Model comparison can then be performed by investigating the ratio of the likelihoods of two alternative models (Table 1). We have adopted the simple likelihood approach (versus a full-fledged Bayesian analysis) because the number of fit parameters are small and equal for each model, the models are simple and low-dimensional, and we have little prior information about the parameters. See Fig. S3 and Table S2 in File S1 for a Bayesian analysis and comparison. Note that parameters (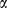, 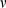) obtained from likelihood maximization of this model will be equivalent to those obtained by performing least-squares regression for the stated models on the logarithms of the folding times. Our maximum likelihood approach provides an estimate of the width of the fits involved (in the form of 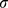) and also a mechanism for rigorous model comparison via likelihood ratios.

**Table 1:** Likelihood Ratios of 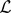-Maximized Models.

Model ^1^	Pr. Law	Exp.	S. E. 1/2	S. E. 2/3	Null
Power Law		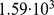	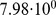	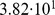	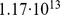
Exponential	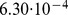		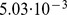	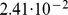	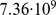
S. E. 1/2	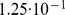	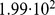		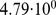	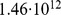
S. E. 2/3	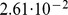	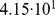	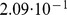		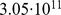
Null	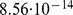	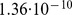	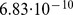	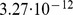	

Primary model is on the left, alternate model along the top - thus, a larger number favors the model in the leftmost column.

### Indirect Method: Biological Limits

We postulate that there exists a critical time, 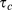, that places a biological upper bound on folding times. Specifically, if a protein folds slower than this time (*i.e.* 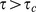) then that protein will be much more likely to aggregate during the course of folding, and therefore is evolutionarily selected against.

The majority of biologically observed proteins should have folding times less than 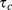, but we postulate that some proteins will have *greater* times. These proteins are those that receive help folding from chaperones or other cellular machinery. It has been estimated that about 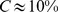 of proteins fall into this category [25].

**Figure 1:**
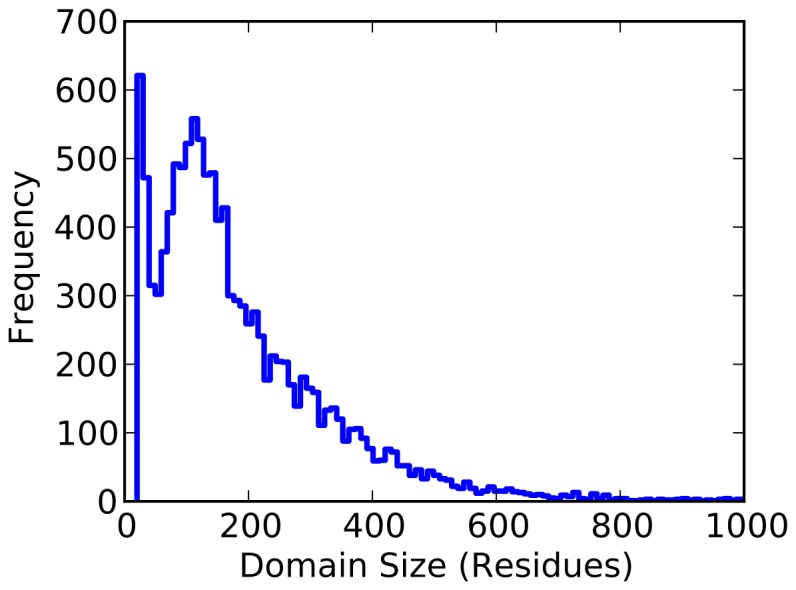
The size distribution of non-homologous protein domains listed in the PDB. Of the 12,151 sequences reported, 39 are larger than 1000 residues (0.32%) - while not shown here for clarity, they were included in subsequent analyses. Data were obtained from the NIH's VAST algorithm (http://www.ncbi.nlm.nih.gov/Structure/VAST/nrpdb.html) on 5/5/2012 with a dissimilarity p-value of 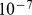.

Together, these assumptions allow us to build a model for the predicted distribution of protein chain lengths. The size distribution of domains (Fig. 1) can be roughly approximated by a Gaussian with parameters 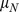 and 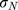. In that case,
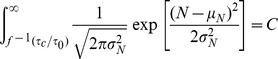
(3)where 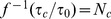 is the chain length corresponding to 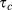 for a specific model (power law, exponential, etc.), and 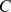 is the percentage of proteins with folding times slower than 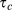.

This framework is, of course, an approximation. There are undoubtedly many other factors affecting the optimal sizes of proteins beyond merely their folding times. Metabolic efficiency, structural packing constraints [26], [27], and the behavior of specific proteins in their local cellular environments certainly play a role. Nonetheless, the concept of an upper limit to the folding times is reasonable, and our aim here is to simply extract some general comments about the reasonableness of predicted folding times, rather than make quantitatively accurate predictions.

## Results and Discussion

**Figure 2:**
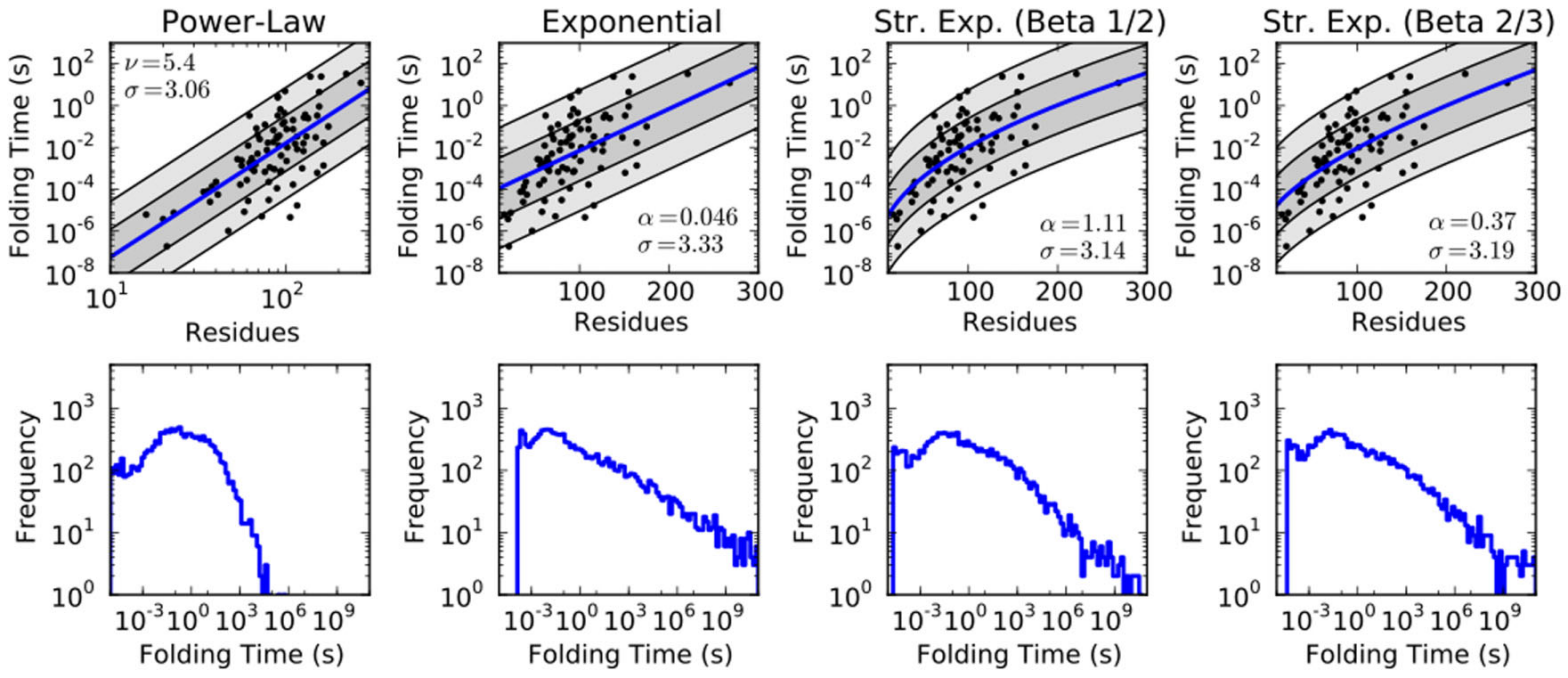
The predicted models for the folding rate law, overlaid with measurements of folding times (top), and the putative folding time distributions these models imply (bottom). Parameter values derived from a maximum likelihood fit are displayed, along with intervals indicating the spread in the fit probability distribution. Dark grey shading indicates a factor of 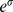, while light grey indicates 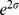.

Direct fitting of all proposed models to the available data yields reasonable results for each (Fig. 2). Each model reports a scale parameter (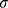) of approximately 3, which indicates that 68% of proteins will have folding times within a factor of 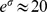 from the time predicted by the rate law, and 95% will be within a factor of 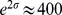. Thus, with knowledge of only the chain length alone, one can estimate the folding time of a protein to within about 2 orders of magnitude and be correct 95% of the time. Since the available data spans folding times of more than 9 orders of magnitude (between 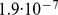 and 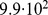 seconds), this demonstrates that chain length captures much of the variation in folding times.

To further assess the presence or absence of an overall rate-length law the likelihood of a null model, which modeled the logarithm of folding rates as a Gaussian distributed independent of chain length (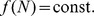), was computed. This null model was found to be significantly less likely (at least a factor of 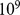 less probable) given the data than any of the four rate-length models proposed (Table 1).

Do the data support any one model? The power-law model is slightly favored by comparing the likelihoods that each model generated the observed data (Table 1). In such comparisons, typically a ratio of 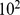 or greater is considered significant, and often models differ by hundreds of orders of magnitude [28] – thus, the power law model is better supported by the data, but only by a modest margin. Further, an attempt to fit the stretched exponential form with 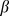 as a variable parameter resulted in an unreasonably small value of 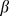 along with a very large value of 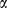, resulting in a fit that is very close to the power law (Fig. S2 in File S1). Finally, the power law model has the smallest fit 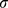, indicating that it explains the most variation in the data, and attributes less to orthogonal factors.

**Figure 3:**
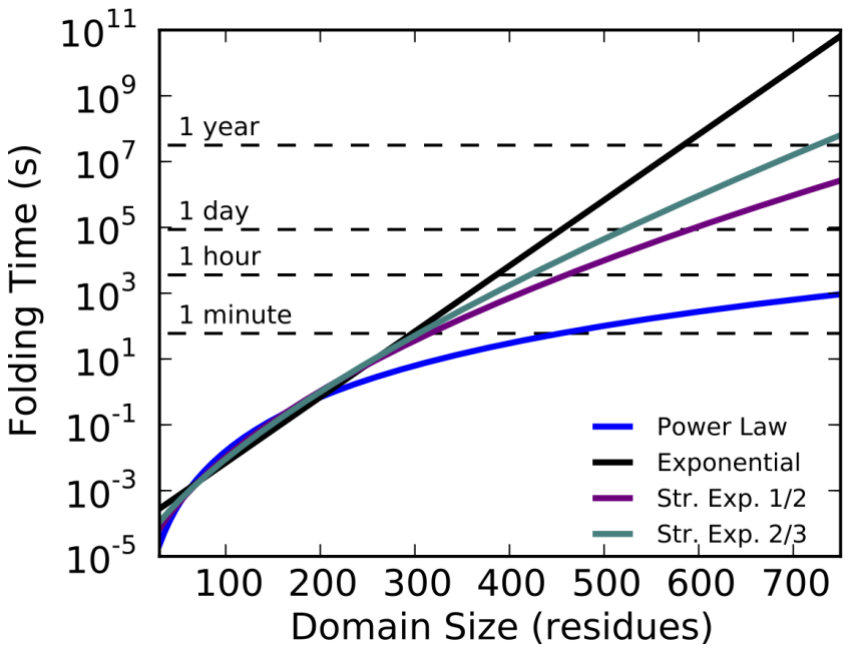
The predicted folding times from each model in Figure 2, in a direct comparison. Intuitive timescales are denoted for clarity.

It is clear, however, that there is little difference between the models in the range of available data. These models diverge significantly only for very large proteins (Fig. 3). We conclude that, with given experimental data, a direct statistical analysis cannot support any one of the theoretically proposed rate-length laws.

Despite this, each law yields significantly different predictions for the distribution of folding times, generated by transforming the known distribution of domain sizes into each of the different models (Fig. 2). The most significant differences are in the tails of these distributions, where the exponential forms predict much longer folding times for the largest proteins (Table 2). The power law model predicts no proteins fold in times longer than an hour, while the exponential forms show a significant number of proteins with folding times longer than a day (Fig. 3).

**Table 2:** Estimated Fraction of Protein Domains with Folding Times Greater than Time Indicated.

	Hour	Day	Month	Year
Power Law	0.41%	0.01%	0.00%	0.00%
Exponential	9.56%	5.70%	3.34%	2.46%
S. E. 1/2	5.48%	2.37%	0.95%	0.57%
S. E. 2/3	7.49%	3.53%	1.74%	1.11%

An evaluation of the reasonableness of these folding time distributions is provided by the critical time 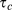 for each model (Table 3). For reasonable values of 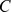 (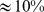, predicted from experiment [25]), the power law 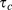 is on the order of minutes. The exponential forms, on the other hand, predict 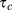 is on the order of hours. Compare this to some cellular benchmarks, as done by Rollins and Dill (personal communication): the 16 second average time to synthesize a protein in *E. Coli* (325 residues×0.05 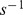 synthesis rate) [29], the 30 seconds it takes for GroEL to refold a protein [30], [31], or the 20 min *E. Coli* doubling time.

**Table 3:** Most Likely 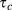 for Values of 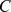 (in seconds).

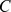	0.1	0.05	0.01	0.001
Power Law	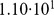	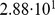	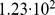	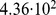
Exponential	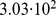	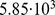	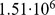	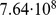
S. E. 1/2	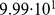	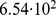	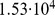	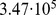
S. E. 2/3	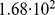	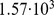	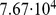	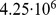

**Figure 4:**
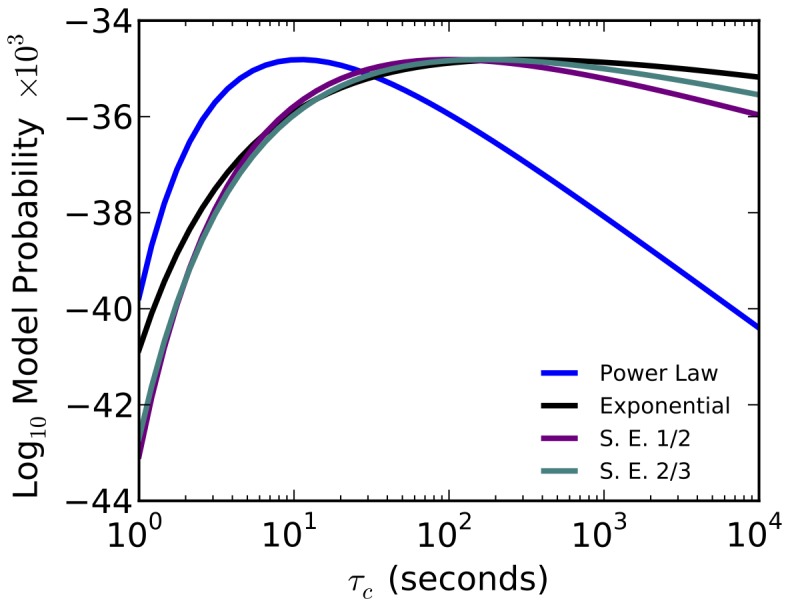
The probability that each of the rate-length laws reproduces the experimentally observed domain size distribution given a value of 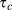. Given a rate-length law mapping chain length to folding times, if the distribution of sizes of protein domains is modeled as a Gaussian, choosing a timescale 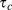 above which proteins need assistance to fold, and an approximate fraction 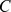 of proteins that need assistance to fold fully specifies the distribution of domain sizes (eq. 3; here, 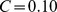). That distribution was used as a model for the generation of the empirically observed domain size distribution reported in Fig. 1,. Here, we plot the probability that the empirically observed distribution resulted from the model indicated. Note the y-axis is 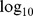 and divided by 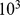 for clarity, so small differences on the plot are actually quite large.

Indeed, picking a reasonable value of 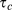 and calculating the probability of observing the empirically observed domain size distribution (Fig. 4) shows that for values of 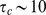 seconds, the power law model is clearly the best. However, for any values of 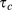 greater than 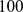 seconds, the exponential laws are much better models.

## Conclusions

We conclude that while chain length is a statistically significant factor for predicting folding rates, current experimental data do no support any one of the proposed models for the rate-length relationship over any other. While the power law model appears to best explain the available raw data, it results in very fast predicted folding times. The exponential forms, while doing a marginally poorer job of explaining the raw data, yield a distribution of folding times more in line with what we expect from biology. Given the current available data, no clear victor emerges.

Previous theories have claimed that simply predicting one of the four laws investigated here is strong evidence in support of that theory. This is manifestly not the case – not only must the proposed law be reasonable, but it must also predict reasonable parameter estimates, and even then the supporting evidence the rate scaling law can provide given current data is limited. Conversely, the analytical theories mentioned here are not ruled out by the current available data. This is most striking in the case of the exponential form, since exponential scaling of the folding times has often been associated with Levinthal's paradox. This study shows that exponential scaling is reasonable given current experimental data, so long as the exponential scaling constant (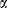) is sufficiently small.

Clear evidence for any one rate law remains missing. However with a few clear examples of very large globular proteins (500 residues or larger) capable of folding unassisted *in vitro*, it might be possible to discriminate between the models proposed here. Figure 3 clearly shows the divergences between predicted folding times for large proteins, and shows how just a few data points in this extreme regime might be able to begin differentiating between the proposed models investigated here.

## Supporting Information

File S1
**Supplemental Information.** **Figure S1.** The rate-length data from each source used, plotted together. Even though there is some overlap in reported sequences, many identical proteins have different chain lengths or folding times reported. We combined these data for our analysis, eliminating only identical measurements. **Figure S2.** The parameter fit for a stretched exponential, 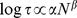, with 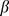 a free parameter. Notice how the fit is perfectly straight on a log-log plot, a characteristic trait of power laws. **Figure S3.** The parameter posteriors for each model are sharply peaked around their modes. Plotted here is the maximum (not the marginal value) of the posterior at various values of the key parameters 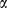 or 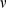. One can see that the likelihood has a sharply peaked value along this dimension. **Table S1.** The KineticDB dataset with mutants included. Since there are only a few proteins with mutants, and there are many mutants for these few proteins, this database gives artificially more weight to those individual proteins. **Table S2.** Bayes factors comparing datasets.(PDF)
