# Supplementary material for: Inferring the Rate-Length Law of Protein Folding
Source: PLoS One. 2013 Dec 5;8(12):e78606. doi: 10.1371/journal.pone.0078606 (PMC3855161; doi:10.1371/journal.pone.0078606)
Supplement: File S1 — Supplemental Information. Figure S1. The rate-length data from each source used, plotted together. Even though there is some overlap in reported sequences, many identical proteins have different chain lengths or folding times reported. We combined these data for our analysis, eliminating only identical measurements. Figure S2. The parameter fit for a stretched exponential, , with a free parameter. Notice how the fit is perfectly straight on a log-log plot, a characteristic trait of power laws. Figure S3. The parameter posteriors for each model are sharply peaked around their modes. Plotted here is the maximum (not the marginal value) of the posterior at various values of the key parameters or . One can see that the likelihood has a sharply peaked value along this dimension. Table S1. The KineticDB dataset with mutants included. Since there are only a few proteins with mutants, and there are many mutants for these few proteins, this database gives artificially more weight to those individual proteins. Table S2. Bayes factors comparing datasets. (PDF) [file pone.0078606.s001.pdf]

Supplemental Information: Inferring the Rate-Length Law of Protein Folding

Thomas J. Lane[1], Vijay S. Pande[2,*]
1 Department of Chemistry, Stanford University, Stanford, CA, USA
2 Departments of Chemistry, Computer Science, and Biophysics, Stanford University, Stanford, CA, USA
∗ **E-mail: pande@stanford.edu**

Different Datasets of Protein Folding Kinetic Data

We are aware of four collections of protein folding kinetic data, here termed "KineticDB" [1], "Liang" [2], "Muñoz" [3], "Finkelstein" [4]; note Finkelstein is reportedly a subset of KineticDB. Each is purported to be extracted directly from the primary literature. Interestingly, while there is much overlap of reported proteins between each dataset, there are systematic inconsistencies between them. Most report that they extrapolate the folding times to zero denaturant – we suspect this is the origin of the discrepancy, but have not undertook a detailed investigation. Instead, we chose the KineticDB dataset because it contained the most proteins, appeared well curated, and restricted entries to proteins at zero denaturant, near room temperature, and at neutral pH.

Interestingly, despite their discrepancies, the datasets appear to be similar on a coarse level (Fig. S1). Further, our fitting analysis run on each independently results in parameters that are not too far from one another (Table S1), and the rest of our results (which primarily follow from these parameters) are very similar between datasets.

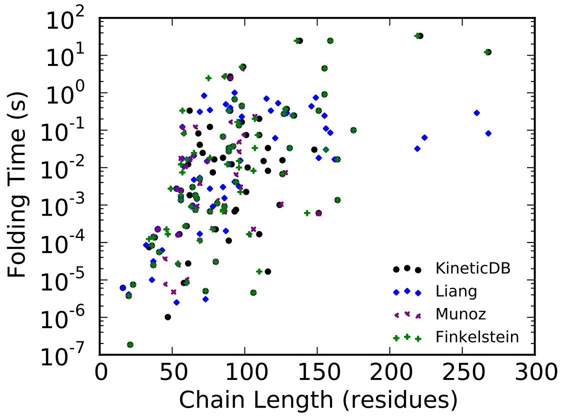

Figure 1. The rate-length data from each source used, plotted together. Even though there is some overlap in reported sequences, many identical proteins have different chain lengths or folding times reported. We combined these data for our analysis, eliminating only identical measurements.

For this study, we simply extracted all non-mutant entries from the KineticDB and employed their reported chain lengths and folding times in water.

Table 1. Parameters Estimated from Different Datasets

	Pr. Law		Expon.		S. E. 1/2		S. E. 2/3	
Dataset	ν	σ	α	σ	α	σ	α	σ
KineticDB	5.44	3.06	0.046	3.32	1.11	3.13	0.37	3.19
KDB (mut) [1]	4.46	2.42	0.040	2.53	0.91	2.45	0.31	2.47
Liang	4.75	2.54	0.041	2.90	0.94	2.70	0.31	2.77
Munoz	4.79	3.05	0.054	3.14	1.04	3.09	0.37	3.11
Finkelstein	5.24	3.08	0.048	3.32	1.08	3.17	0.36	3.22

Stretched Exponential with β as a Free Parameter

In the main text we investigated the specific stretched exponential forms proposed by extant simple models (*i.e.* with β as 1/2 and 2/3). There is no reason why β cannot be simply fit as a free parameter, however, and we have performed this fit (Fig. S2). Remarkably, the most likely parameters are those

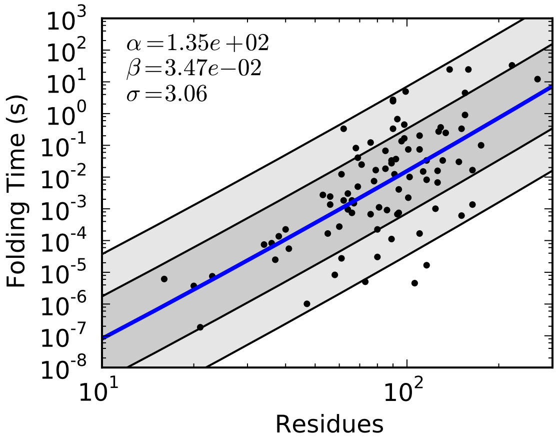

Figure 2. The parameter fit for a stretched exponential, $\log \tau \propto \alpha N^{\beta}$, with β a free parameter. Notice how the fit is perfectly straight on a log-log plot, a characteristic trait of power laws.

with $\alpha \approx 10^2$ and $\beta \approx 10^{-2}$, which is far outside the range predicted by theory. With these extreme parameters, the model appears to be a power law form over the range of fit data, and is linear on a log-log plot to about 10^{25} residues. This simply verifies that the data are best explained by a power law, and demonstrates that the power law fit with free β is sufficiently flexible to capture this fact.

Bayesian Model Comparison

As mentioned in the main text, it is entirely possible to pursue a full-fledged Bayesian analysis to determine the parameters for each model. Here, we show the commonalities and differences between the maximum likelihood approach pursued in the main text and a Bayesian approach. We show that it makes little difference which is chosen.

We have little *a priori* information about the parameters we are to fit besides the fact that each (α, ν, σ) must be greater than zero. Thus, we choose a uniform prior for each over the interval $[0, \infty]$, and

write this distribution $\pi(\theta)$, where θ will stand in for the vector of parameters relevant to a particular model. Then we can write the Bayesian *posterior* as

$$P(\theta|D) \propto \mathcal{L}(D|\theta)\pi(\theta)$$

with $\mathcal{L}$ the likelihood from the main text. Since π is uniform, however, we can write

$$P(\theta|D) \propto \mathcal{L}(D|\theta)$$

as long as all the parameters are in the interval $[0, \infty]$. Given the posterior $P(\theta|D)$, it is common to simply take the mode as the best representative set of parameters - which are the maximum likelihood parameters used in the main manuscript. This choice is justified because we have found the posterior to be strongly peaked around the mode, as seen in Figure S3.

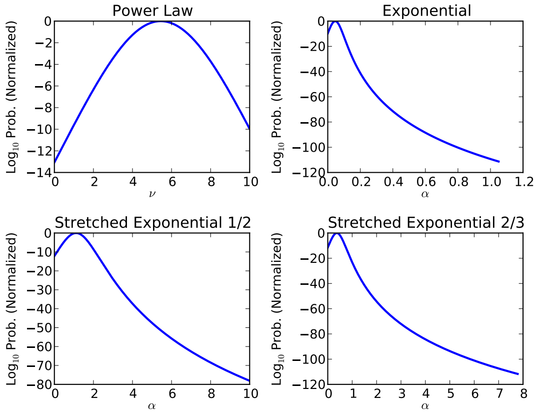

Figure 3. The parameter posteriors for each model are sharply peaked around their modes. Plotted here is the maximum (not the marginal value) of the posterior at various values of the key parameters α or ν. One can see that the likelihood has a sharply peaked value along this dimension.

Table 2. Bayes Factors Comparing Datasets

	Pr. Law	Exp.	S. E. 1/2	S. E. 2/3	Null
Power Law		$1.32 \cdot 10^{5}$	$3.55 \cdot 10^{1}$	$4.74 \cdot 10^{2}$	$8.93 \cdot 10^{12}$
Exponential	$7.55 \cdot 10^{-6}$		$2.68 \cdot 10^{-4}$	$3.58 \cdot 10^{-3}$	$6.74 \cdot 10^{7}$
S. E. 1/2	$2.81 \cdot 10^{-2}$	$3.73 \cdot 10^{3}$		$1.33 \cdot 10^{1}$	$2.51 \cdot 10^{11}$
S. E. 2/3	$2.11 \cdot 10^{-3}$	$2.80 \cdot 10^{2}$	$7.50 \cdot 10^{-2}$		$1.88 \cdot 10^{10}$
Null	$1.12 \cdot 10^{-13}$	$1.48 \cdot 10^{-8}$	$3.98 \cdot 10^{-12}$	$5.31 \cdot 10^{-11}$	

The only other difference between a Bayesian analysis and the likelihood methods are the way models are compared. In contrast to a likelihood ratio, Bayesian statistics recommends a Bayes' factor $\mathcal{F}$ [5], which compares two models (M_1, M_2)

$$\mathcal{F}_{12} = \frac{\int_\theta \mathcal{L}_{M_1}(D|\theta)\pi_{M_1}(\theta)}{\int_\theta \mathcal{L}_{M_2}(D|\theta)\pi_{M_2}(\theta)}$$

which explicitly includes information from all possible values of the parameters. The likelihood ratios used in the main text are a saddle approximation to the integrals, and thus these two methods match in

the case where the posteriors are highly peaked. Table S2 shows the calculated Bayes' factors for each model. These Bayes' factors were calculated by also integrating over τ_0, the parameter accounting for a constant offset (or units) in our fits. The domain of integration for τ_0 was restricted to $[-50, 10]$ for numerical stability - increasing it beyond this range resulted in little difference.

Details of the Gaussian Fit Model

Here we go over some of the details of the Gaussian model that was used to predict the folding time distribution. In the main text, we presented the central equation

$$\int_{f^{-1}(\tau_c/\tau_0)}^{\infty} \frac{1}{\sqrt{2\pi\sigma_N^2}} \exp\left[\frac{(N-\mu_N)^2}{2\sigma_N^2}\right] = C$$

from which all of our subsequent analysis begins. Using this model, we can predict τ_c for a chosen model and chosen C. To do this, μ_N was fixed at 105, the empirical mode of the distribution of domain sizes (main text Fig. 1) – this appeared to make subsequent fits more robust. The parameter σ_N was then fit, via likelihood maximization, to the same empirical distribution. Subsequently, a series of values of C were chosen, and these implied values of τ_c for each model, resulting in main text Table 3.

It is also possible to calculate the likelihood of observing the known empirical data given a specific τ_c, via likelihood maximization. The procedure here was straightforward; first, C was set to 0.10, μ_N was again fixed at 105, τ_c was fixed at an arbitrary value for a chosen model, and σ_N was varied to maximize the likelihood of witnessing the empirical distribution given that model. The results for a scan of τ_c for each model is reported in Figure 4 in the main text.

References

1. Bogatyreva NS, Osypov AA, Ivankov DN (2009) KineticDB: a database of protein folding kinetics. Nucleic Acids Research 37: D342–D346.

2. Ouyang Z, Liang J (2008) Predicting protein folding rates from geometric contact and amino acid sequence. Protein Sci 17: 1256–1263.

3. De Sancho D, Muñoz V (2011) Integrated prediction of protein folding and unfolding rates from only size and structural class. Phys Chem Chem Phys 13: 17030.

4. Ivankov DN, Finkelstein AV (2010) Protein Folding as Flow across a Network of FoldingUnfolding Pathways. 2. The "In-Water" Case. J Phys Chem B 114: 7930–7934.

5. Kass RE, Rafterty AE (1995) Bayes Factors. J Am Stat Assoc 90: 773–795.
